# Supplementary material for: Surface association sensitizes Pseudomonas aeruginosa to quorum sensing
Source: Nat Commun. 2019 Sep 11;10:4118. doi: 10.1038/s41467-019-12153-1 (PMC6739362; doi:10.1038/s41467-019-12153-1)
Supplement: Supplementary file 1 — Supplementary Information [file 41467_2019_12153_MOESM1_ESM.pdf]

Supplementary Information for

Surface association sensitizes *Pseudomonas aeruginosa* to quorum sensing

Chuang et al.

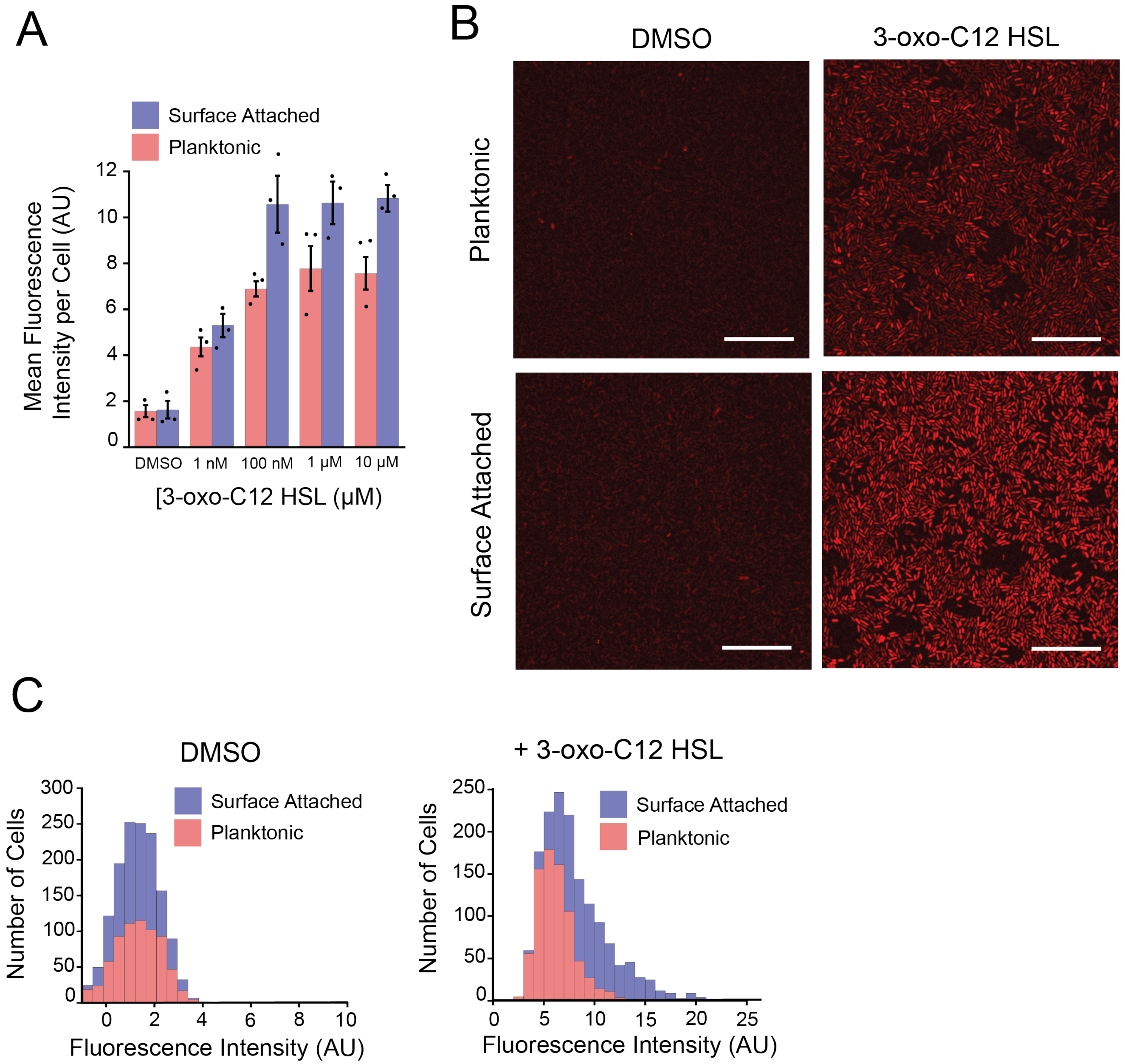

Supplementary Figure 1. (A) Expression of P_{lasI}-*mCherry* promoter fusion in surface-attached and planktonic *ΔlasI P. aeruginosa* supplemented with various concentrations of 3-oxo-C12 HSL. Cultures were transferred to glass-bottom dishes when OD_{600nm} reached 0.5-0.6, and grown for 1 h to allow for surface attachment. Mean fluorescent intensity per cell was calculated for 500-1000 cells, and values shown are averages of three independent experiments. Error

bars represent standard error. B) Representative images of samples in (A) treated with 10 µM 3OC12 or DMSO (scale bars = 20 µm). C) Distribution of single-cell fluorescent intensity of samples in (A) treated with 10 µM 3OC12 or DMSO. Source data are provided as a Source Data file.

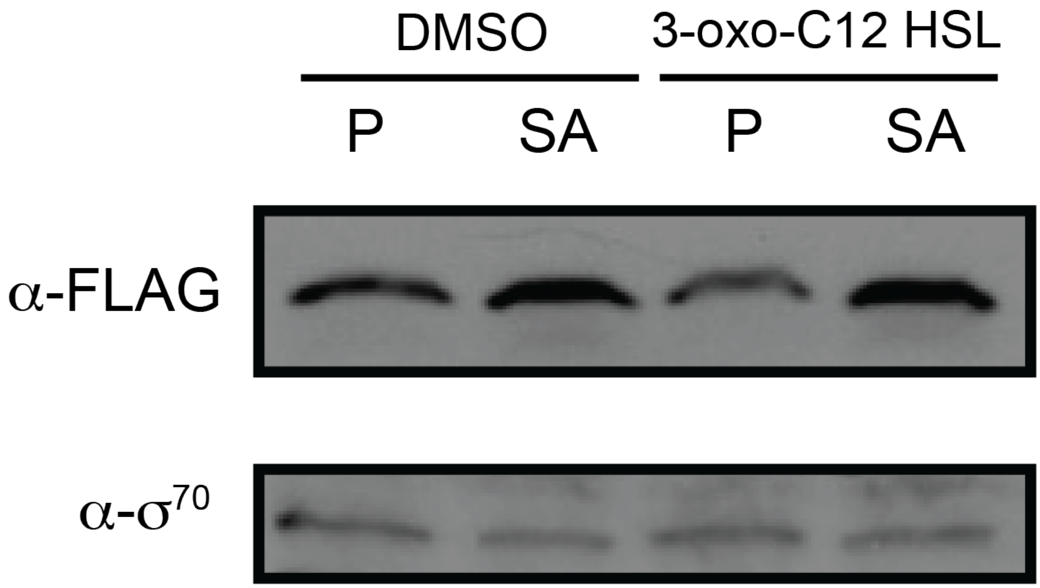

Supplementary Figure 2. Western blot analysis of FLAG-LasR protein levels in surface-attached and planktonic *P. aeruginosa*. Cultures were transferred to polystyrene dishes when cell density reached OD_{600nm} 0.5-0.6,, and incubated for 1 h to allow for surface attachment. Equal amounts of and surface-attached and planktonic cells, based on OD_{600nm}, were separated by SDS-PAGE and probed with an anti-FLAG monoclonal antibody and anti-σ^{70} as a loading control. Experiments were performed in triplicate, and similar trends were observed across all three independent experiments.

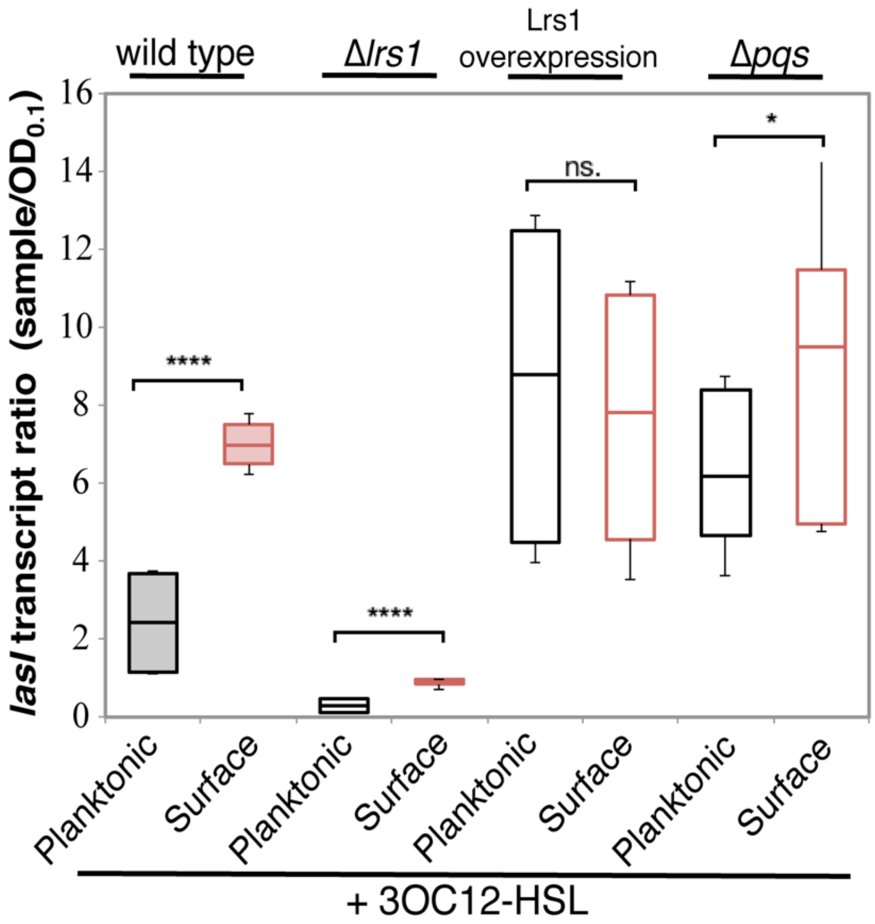

Supplementary Figure 3. *lasI* mRNA levels in wild type, Δ*lrs1*, Lrs1 overexpression and, Δ*pqsA-E* strain from qRT-PCR. Error bars in plots represent standard deviation and boxes indicate 25/75 data with the center representing the mean. A two-tailed student's t-test performed to determine significance between samples (n = 9). Source data are provided as a Source Data file.

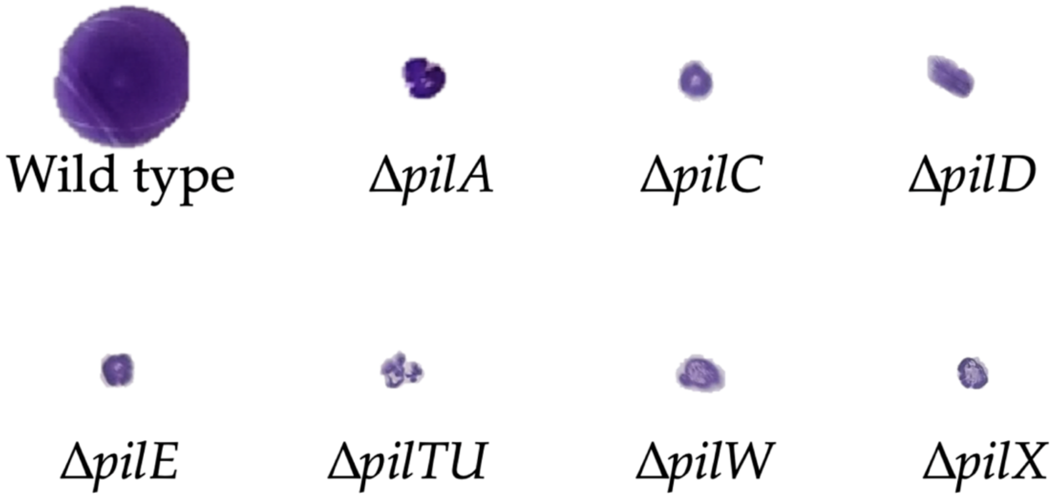

Supplementary Figure 4. Twitching phenotypes for pilin mutants Δ*pilA*, Δ*pilC*, Δ*pilD*, Δ*pilE*, Δ*pilTU* Δ*pilW*, and Δ*pilX*.

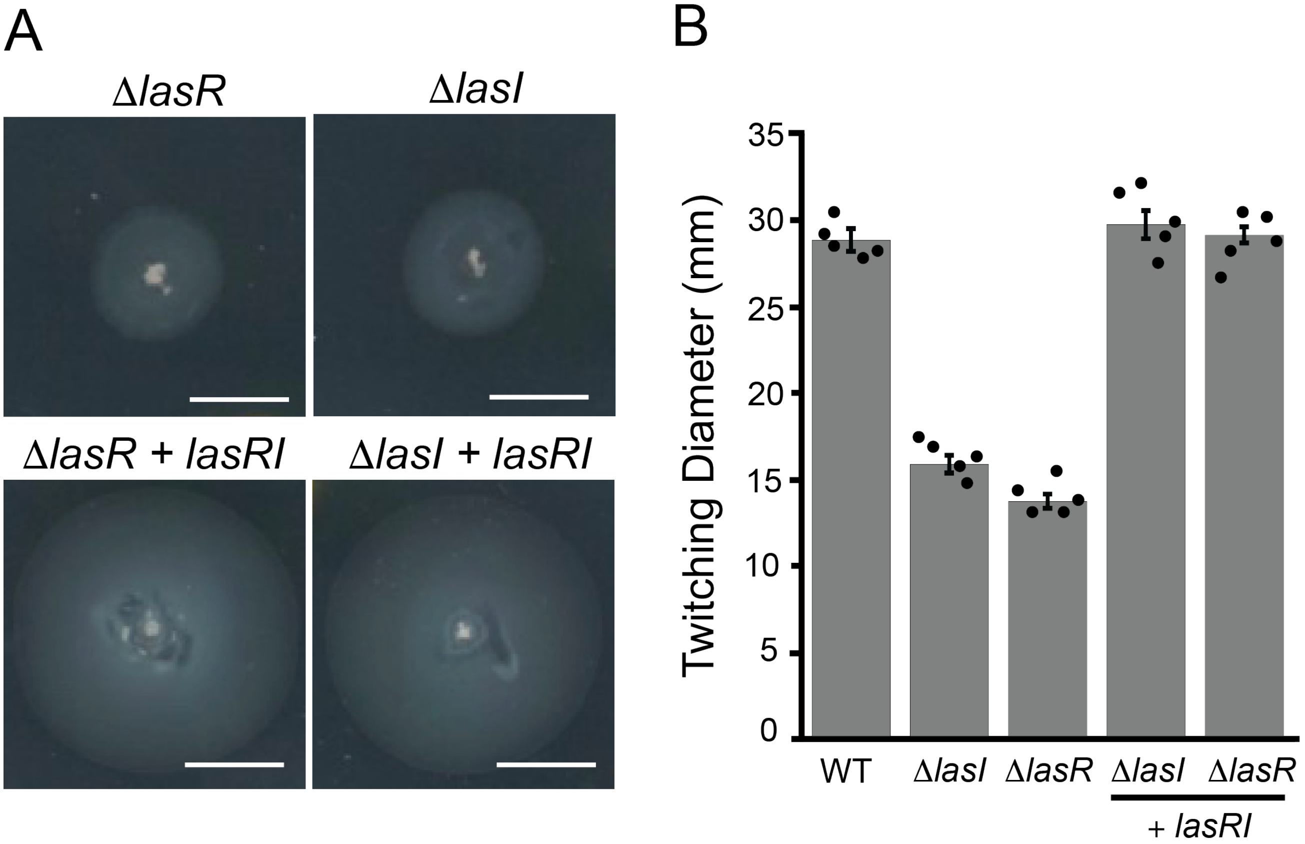

Supplementary Figure 5. (A) Restoration of twitching motility of *lasR* and *lasI* mutants by complementation (scale bars = 10 mm). (B) Quantification of twitching zones for strains in (A). Values are averages (n = 5) and error bars represent standard error. Source data are provided as a Source Data file.
