# Supplementary material for: Surface association sensitizes Pseudomonas aeruginosa to quorum sensing
Source: Nat Commun. 2019 Sep 11;10:4118. doi: 10.1038/s41467-019-12153-1 (PMC6739362; doi:10.1038/s41467-019-12153-1)
Supplement: Supplementary file 3 — Description of Additional Supplementary Files [file 41467_2019_12153_MOESM3_ESM.pdf]

Description of Additional Supplementary Files

File Name: Supplementary Data 1
Description: Surface response of LasR regulon genes. Values indicate the ratio of surface to planktonic cells of LasR regulon genes (48) at 3 hours of surface-association. Student's t-test P values < 0.1 are highlighted in dark green.
