# Supplementary material for: Surface association sensitizes Pseudomonas aeruginosa to quorum sensing
Source: Nat Commun. 2019 Sep 11;10:4118. doi: 10.1038/s41467-019-12153-1 (PMC6739362; doi:10.1038/s41467-019-12153-1)
Supplement: Supplementary file 5 — Reporting Summary [file 41467_2019_12153_MOESM5_ESM.pdf]

nature research

Corresponding author(s): Chuang S., Vrla G., Froelich K., Gitai Z.

Last updated by author(s): Aug 12, 2019

Reporting Summary

Nature Research wishes to improve the reproducibility of the work that we publish. This form provides structure for consistency and transparency in reporting. For further information on Nature Research policies, see Authors & Referees and the Editorial Policy Checklist.

Statistics

For all statistical analyses, confirm that the following items are present in the figure legend, table legend, main text, or Methods section.

n/a	Confirmed	
☐	☒	The exact sample size (*n*) for each experimental group/condition, given as a discrete number and unit of measurement
☐	☒	A statement on whether measurements were taken from distinct samples or whether the same sample was measured repeatedly
☐	☒	The statistical test(s) used AND whether they are one- or two-sided<br>*Only common tests should be described solely by name; describe more complex techniques in the Methods section.*
☒	☐	A description of all covariates tested
☒	☐	A description of any assumptions or corrections, such as tests of normality and adjustment for multiple comparisons
☒	☐	A full description of the statistical parameters including central tendency (e.g. means) or other basic estimates (e.g. regression coefficient) AND variation (e.g. standard deviation) or associated estimates of uncertainty (e.g. confidence intervals)
☐	☒	For null hypothesis testing, the test statistic (e.g. *F*, *t*, *r*) with confidence intervals, effect sizes, degrees of freedom and *P* value noted<br>*Give P values as exact values whenever suitable.*
☒	☐	For Bayesian analysis, information on the choice of priors and Markov chain Monte Carlo settings
☒	☐	For hierarchical and complex designs, identification of the appropriate level for tests and full reporting of outcomes
☒	☐	Estimates of effect sizes (e.g. Cohen's *d*, Pearson's *r*), indicating how they were calculated

Our web collection on statistics for biologists contains articles on many of the points above.

Software and code

Policy information about availability of computer code

Data collection	no software was used
Data analysis	Sequence files from the facility were processed with customized Python scripts from our lab and aligned to the P. aeruginosa UCBPP-PA14 genome using Bowtie 2. Raw counts were divided by total number of reads per sample and converted to counts per million (CPM) for comparison.

For manuscripts utilizing custom algorithms or software that are central to the research but not yet described in published literature, software must be made available to editors/reviewers. We strongly encourage code deposition in a community repository (e.g. GitHub). See the Nature Research guidelines for submitting code & software for further information.

Data

Policy information about availability of data

All manuscripts must include a data availability statement. This statement should provide the following information, where applicable:

- Accession codes, unique identifiers, or web links for publicly available datasets
- A list of figures that have associated raw data
- A description of any restrictions on data availability

Sequencing data that support the findings of this study have been deposited in SRA at NCBI with the links to the following accession codes below
https://www.ncbi.nlm.nih.gov/biosample/11250570
https://www.ncbi.nlm.nih.gov/biosample/11250571
https://www.ncbi.nlm.nih.gov/biosample/11250572
https://www.ncbi.nlm.nih.gov/biosample/11250573
https://www.ncbi.nlm.nih.gov/biosample/11250574
https://www.ncbi.nlm.nih.gov/biosample/11250575
https://www.ncbi.nlm.nih.gov/biosample/11250576

https://www.ncbi.nlm.nih.gov/biosample/11250577
https://www.ncbi.nlm.nih.gov/biosample/11250578
https://www.ncbi.nlm.nih.gov/biosample/11250579
https://www.ncbi.nlm.nih.gov/biosample/11250580
https://www.ncbi.nlm.nih.gov/biosample/11250581
https://www.ncbi.nlm.nih.gov/biosample/11250582
https://www.ncbi.nlm.nih.gov/biosample/11250583
https://www.ncbi.nlm.nih.gov/biosample/11250584
https://www.ncbi.nlm.nih.gov/biosample/11250585
https://www.ncbi.nlm.nih.gov/biosample/11250586
https://www.ncbi.nlm.nih.gov/biosample/11250587

Field-specific reporting

Please select the one below that is the best fit for your research. If you are not sure, read the appropriate sections before making your selection.

☒ Life sciences ☐ Behavioural & social sciences ☐ Ecological, evolutionary & environmental sciences

For a reference copy of the document with all sections, see nature.com/documents/nr-reporting-summary-flat.pdf

Life sciences study design

All studies must disclose on these points even when the disclosure is negative.

Sample size	Each condition assayed was completed with three biological replicates. qRT-pcr data was collected with three technical replicates of each biological replicate, for a total of n = 9.
Data exclusions	*Describe any data exclusions. If no data were excluded from the analyses, state so OR if data were excluded, describe the exclusions and the rationale behind them, indicating whether exclusion criteria were pre-established.*
Replication	three biological replicates
Randomization	*Describe how samples/organisms/participants were allocated into experimental groups. If allocation was not random, describe how covariates were controlled OR if this is not relevant to your study, explain why.*
Blinding	*Describe whether the investigators were blinded to group allocation during data collection and/or analysis. If blinding was not possible, describe why OR explain why blinding was not relevant to your study.*

Reporting for specific materials, systems and methods

We require information from authors about some types of materials, experimental systems and methods used in many studies. Here, indicate whether each material, system or method listed is relevant to your study. If you are not sure if a list item applies to your research, read the appropriate section before selecting a response.

Materials & experimental systems

n/a	Involved in the study	
☒	☐	Antibodies
☒	☐	Eukaryotic cell lines
☒	☐	Palaeontology
☒	☐	Animals and other organisms
☒	☐	Human research participants
☒	☐	Clinical data

Methods

n/a	Involved in the study	
☒	☐	ChIP-seq
☒	☐	Flow cytometry
☒	☐	MRI-based neuroimaging
